# Combination of BFHY with Cisplatin Relieved Chemotherapy Toxicity and Altered Gut Microbiota in Mice

**DOI:** 10.1155/2023/3568416

**Published:** 2023-05-19

**Authors:** Yuan Feng, Ying Jiang, Ying Zhou, Zhan-hua Li, Qi-qian Yang, Jin-feng Mo, Yu-yan Wen, Li-ping Shen

**Affiliations:** ^1^Department of Respiratory Medicine, Ruikang Hospital Affiliated to Guangxi University of Traditional Chinese Medicine, Nanning, 530011 Guangxi, China; ^2^Department of Neurology, Ruikang Hospital Affiliated to Guangxi University of Traditional Chinese Medicine, Nanning, 530011 Guangxi, China; ^3^Department of Radiation Oncology, Ruikang Hospital Affiliated to Guangxi University of Traditional Chinese Medicine, Nanning, 530011 Guangxi, China

## Abstract

**Aim:**

We sought to profile gut microbiota's role in combination of Bu Fei Hua Yu (BFHY) with cisplatin treatment.

**Methods:**

Non-small cell lung cancer (NSCLC) mice model were constructed followed by treatment with cisplatin alone or combined with BFHY. Mice weight and tumor volume were measured during the experiment. And mice cecum were detected by hematoxylin and eosin, cecum contents were collected for Enzyme Linked ImmuneSorbent Assay, and stool were profiled for metagenomic sequencing.

**Results:**

Combination of BFHY with cisplatin treatment decreased the tumor growth and relieved the damage of cecum. Expressions of interleukin-6 (IL-6), interleukin-1*β* (IL-1*β*), monocyte chemotactic protein 1 (MCP), and interferon-*γ* (IFN-*γ*) were decreased compared with cisplatin treatment alone. Linear discriminant analysis effect size analysis showed that *g_Parabacteroides* was downregulated and *g_Escherichia* and *g_Blautia* were upregulated after cisplatin treatment. After combination with BFHY, *g_Bacteroides* and *g_Helicobacter* were decreased. *g_Klebsiella*, *g_Unclssified_Proteobacteria*, and *g_Unclssified_Clostridiates* were increased. Moreover, heatmap results showed that *Bacteroides* abundance was increased significantly after cisplatin treatment; BFHY combination treatment reversed this state. Function analysis revealed that multiple functions were slightly decreased in cisplatin treatment alone and increased significantly after combination with BFHY.

**Conclusion:**

Our study provided evidence of an efficacy of combination of BFHY with cisplatin on treatment of NSCLC and revealed that gut microbiota plays a role in it. The above results provide new ideas on NSCLC treatment.

## 1. Introduction

Non-small cell lung cancer (NSCLC) is one of the deadliest cancers in the world [1]. The common therapies for NSCLC contain surgery, radiotherapy, chemotherapy, or targeted therapy, either alone or combination [2]. Biomarker testing is a method to determine the treatment way of patient with NSCLC, although the novel serum biomarkers as well as biomarkers for tumor at present are not enough [3]. However, benefited from advances of the above method, 2-year survival rate for advanced NSCLC patients has been improved [2]. Cisplatin, one of the chemotherapy drugs, is still the efficient way in the first-line management of advanced NSCLC patients, which may cause drug resistance and intestinal toxicity [4].

In recent years, medicinal plants used in traditional Chinese medicines (TCMs) are approved to have the potential as a mainstream form of complementary use for cancer patients in China, and a larger number of TCMs have been used in the treatment of cancers for its effectiveness and lack of serious side effects. In particular, combination of TCM with chemotherapies has the great potential to improve efficacy via multiple molecular mechanisms. For example, Frión-Herrera et al. reported that propolis could resensitize the chemoresistance and induce apoptosis of human colon carcinoma when combined with doxorubicin [5]. Trichostatin A (TSA) is isolated from *Streptomyces hygroscopicus*; Frión-Herrera reported that TSA reduced sunitinib resistance of renal cell carcinoma by triggering intracellular metabolome shits regarding energy metabolism [6]. Bu Fei Hua Yu (BFHY) is a clinical experience formula for treating lung cancer and composed of 12 TCM ingredients. What's more, the ingredients of *Scutellaria barbata*, *Oldenlandia diffusa*, *Astragalus*, *turmeric*, etc., have been proven to have significant resistance tumor effects. Our clinical study showed that combination of BFHY treatment with chemotherapy can significantly improve the clinical efficacy and quality of life by reducing the side effects of intestinal toxicity and can improve the cellular immune function of patients [7, 8]. However, the mechanism involved is not clear.

Gut microbiota, one of the human symbiotic microbial populations, produces vitamins, promotes the metabolization of dietary compounds, resists infiltration of gut pathogens, and affects host's homeostasis through modulating the immune response, inflammation, and metabolism [9, 10]. Among them, gut microbiota has a long-term effect on immune response via producing metabolites or interacting with pattern recognition receptors following with subsequent signaling pathway [10]. Therefore, disrupting the balance of gut microbiota may lead to pathologies, including cancer. Additionally, pathologies and its therapies may affect microbiome changes [9, 11]. However, there are not enough evidences could ascertain that the changes of gut microbiota are the cause or the result [12]. In the progress of chemotherapy, gut microbiota diversity and richness are reduced. In the mucositis rat model, Fijlstra et al. found that the number and diversity of microbiota were decreased during chemotherapy [13]. In addition, Montassier et al. observed that following chemotherapy, the abundances of *Firmicutes* and *Actinobacteria* were significantly decreased, and the abundance of *Actinobacteria* was significantly increased in fecal sample of patients with non-Hodgkin's lymphoma [14]. The above studies indicate that gut microbiota may be a target to improve efficacy of chemotherapy and reduce its toxicity [14, 15]. *Lactobacillus*, *Bifidobacterium*, and *Mycoplasma* enhance the effect of cisplatin through decreasing the levels of oncogenic vascular endothelial growth factor and Ras in lung cancer therapy [12]. Furthermore, increasing evidences have shown that combination of TCM with chemotherapy drugs could enhance the sensitivity of cancer to chemotherapy treatment by restoring the gut microbiota dysbiosis [16, 17].

Based on the above findings, BFHY reduced toxicity of chemotherapy, and gut microbiota had important role in chemotherapy toxicity. Whether BFHY reduced chemotherapy toxicity by regulating gut microbiota is the purpose of our present study. Therefore, NSCLC mice model were constructed first. Then, these mice were treated with cisplatin alone or combined with BFHY. After that, stool were profiled for metagenomic sequencing to evaluate the changes of gut microbiota.

## 2. Methods

### 2.1. Materials and Reagents

The BFHY constituted of *Codonopsis pilosula*, *Astragalus*, *Rehmannia glutinosa*, *Aster tataricus*, *Ligusticum sinense*, *Paeonia anomala*, *Salvia miltiorrhiza*, *Peucedanum praeruptorum*, *Amygdalus Communis Vas*, *Curcuma phaeocaulis Valeton*, *Scirpus fluviatilis*, *Scutellaria barbata*, *Hedyotis diffusa*, *Helicarionidae*, and *Glycyrrhiza uralensis* were acquired from Ruikang Hospital Affiliated to Guangxi University of Chinese Medicine. Cisplatin was purchased from Sigma (P4394-250MG, USA).

### 2.2. Cell Culture and Animals

The human lung carcinoma A549 cells were purchased from CellCook (Guangzhou, China). A549 cells were cultured in F-12K medium (cat: 11875, CellCook) and supplemented with 10% fetal bovine serum. Thirty SPF grade male nude mice (18–20 g) were purchased from Guangdong Medical Laboratory Animal Center and fed in Forevergen Biosciences Co., Ltd. (Guangzhou, China). All mice were adaptively fed for 7 days before experiments. All animal experiments were performed in accordance with the ethics committee of the Forevergen Biosciences Animal Center.

### 2.3. Mice Models of Lung Cancer Construction and Drug Administration

After adaptive feeding, 30 mice were subcutaneously injected with A549 cells at concentration of 5 × 10^6^/mouse in 100 *μ*l. Tumor growth was monitored the other day; when the average tumor volume reached 100–120 mm^3^, the mice were randomly divided into five groups with six each: models of lung cancer (NSCLC), cisplatin treatment (Cis), combination with BFHY low-dose (BFHY_low), combination with BFHY_middle dose (BFHY_middle), and combination with BFHY high-dose (BFHY_high). Mice in Cis groups were administered intraperitoneally with cisplatin (2 mg/kg) every other day for 15 days. Mice in BFHY groups were treated with 2 mg/kg cisplatin (via intraperitoneally injection; every other day) and BFHY of different doses, respectively [via intragastric one time daily; 11.25 g/kg (low), 22.49 g/kg (middle), and 44.98 g/kg (high)] for 15 days [18]. NSCLC and Cis groups were also intragastric with equal volume of phosphate-buffered saline. Mice weight and tumor size were measured every three days, and tumor volumes (mm^3^) were calculated as length × width^2^/2. All mice were euthanized when the cancer volumes in the NSCLC group mice reached about 1000–1500 mm^3^.

### 2.4. H&E Analysis

Hematoxylin and eosin (H&E) staining was performed as previously described [19]. The fresh cecum tissue was collected and fixed in 10% buffered formalin and embedded in paraffin. The prepared paraffin sections (3–5 *μ*m thick) were deparaffinized and hydrated. Then, the slices go through H&E (G1120, Beijing Solarbio Science and Technology Co., Ltd.) in sequence, and finally, the slices were sealed with neutral gum.

### 2.5. ELISA Analysis

The levels of interleukin-6 (IL-6, CSB-E04639m), interleukin-1*β* (IL-1*β*, CSB-E08054m), interferon-*γ* (IFN-*γ*, CSB-E04578m), and monocyte chemotactic protein 1 (MCP-1, CSB-E07430m) in serum and cecum contents were measured by quantitative Enzyme Linked ImmuneSorbent Assay (ELISA) analysis using a microtiter plate reader at 450 nm. The ELISA kits were purchased from Wuhan CUSABIO Co., Ltd.

### 2.6. DNA Extraction, Library Construction, and Sequencing

Mice fecal samples were collected at the end of experiment. Microbial DNA was extracted by using TIANamp Stool DNA Kit (DP328, TIANGEN, Beijing, China) according to manufacturer recommended protocols. Qualified DNA samples (A260/A280 value between 1.6 and 1.8) were randomly broken into fragments in 350 bp length with a Covaris ultrasonic disruptor and after end repair, A-tailing, adapters adding, purification, and polymerase chain reaction amplification for library construction. Accurate quantification (concentration > 3 nM) was used to ensure library quality. The library was pooled and subjected to pair-end sequencing (150 bp) using the Illumina platform (Illumina, San Diego, USA).

### 2.7. Data Preprocessing

Raw data quality-filtered and host contamination removal were performed using Bowtie2 [20] to obtain clean data and then were assembled using MEGAHIT. Gene prediction was implemented by MetaGeneMark with mixed assembled scaftigs for gene catalogue information in each sample. Species (gut microbiota) annotation was starting from the gene catalogue and comparing it with MicroNR library (Version: 2018.01).

### 2.8. Linear Discriminant Analysis Effect Size (LEfSe) Analysis

Rank sum test method was used to analyze the different microbiota between the groups, and the LDA (linear discriminant analysis) was used to achieve dimensionality reduction and evaluate the impact of the different microbiotas, which was the LDA score (LDA > 4).

### 2.9. Functional Analysis of Different Microbiotas between Groups

Unigenes were compared with KEGG database using DIAMOND (blastp, evalue ≤1 × 10^−5^) and filtered (one high-scoring segment pair >60 bits [20]) for subsequent analysis. Microbiota relative abundance of different functional levels was contented. Annotated genes number, relative abundance profile, abundance clustering (Bray–Curtis distance), PCA analysis, and Metastat analysis (*q* value <0.05) of functional differences between groups were implemented.

### 2.10. Statistical Analysis

Multiple *t* test (*p* value <0.05) was performed for mice weight, tumor volume, inflammation factors concentration, and gene numbers between groups by Graph pad prism 8. LEfSe analysis was used for calculating the significantly microbiota between groups with LDA score >4. Hypothesis testing was performed on the functional *p* value then correcting for *q* value (*q* < 0.05) by Metastats [21] for significantly functional pathway between groups.

## 3. Results

### 3.1. BFHY Enhanced the Ability of Cisplatin to Kill Tumors

**Figure 1:**
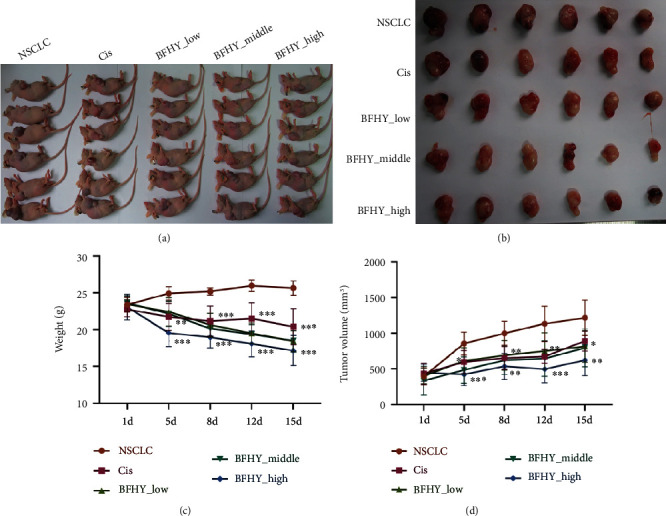
The efficacy of combination of BFHY with cisplatin on mice models of lung cancer. A549 cell lines were subcutaneously injected to nude mouse, when tumor volume reached to 100–120 mm^3^ and divided into five groups randomly then, treated with cisplatin (2 mg/kg) or BFHY (11.25 g/kg, 22.49 g/kg, and 44.98 g/kg) with cisplatin (2 mg/kg) for 15 days. (a) Comparison of mice models of lung cancer and different treatment groups. (b) Comparison of tumor samples excised from all groups. (c) Mice weight was measured at 1, 5, 8, 12, and 15 days after treatment of all groups. (d) Tumor volume (mm^3^) was measured at 1, 5, 8, 12, and 15 days after treatment of all groups. *N* = 6, statistical significance was determined by multiple *t* test, ∗*p* < 0.05, ∗∗*p* < 0.01, ∗∗∗*p* < 0.001.

First, the effect of different dosages of BFHY combined with cisplatin on lung cancer in vivo was studied. The weight of the mice was significantly decreased after cisplatin treatment alone and had a more potent decrease when combined with BFHY_high at the fifth day (Figure 1(a)). Tumor growth measured by size was significantly inhibited by BFHY in a dose-dependent manner; what's more, combination of BFHY_high with cisplatin had the better inhibitory effect on tumor, compared with the cisplatin alone (Figure 1(b)). Final mice weight and tumor size were showed at the end point (Figures 1(c) and 1(d)). The above results indicated that BFHY further strengthened the anti-tumor ability of cisplatin on mice models of lung cancer.

### 3.2. BFHY Relieved Intestinal Damage Caused by Cisplatin Treatment

**Figure 2:**
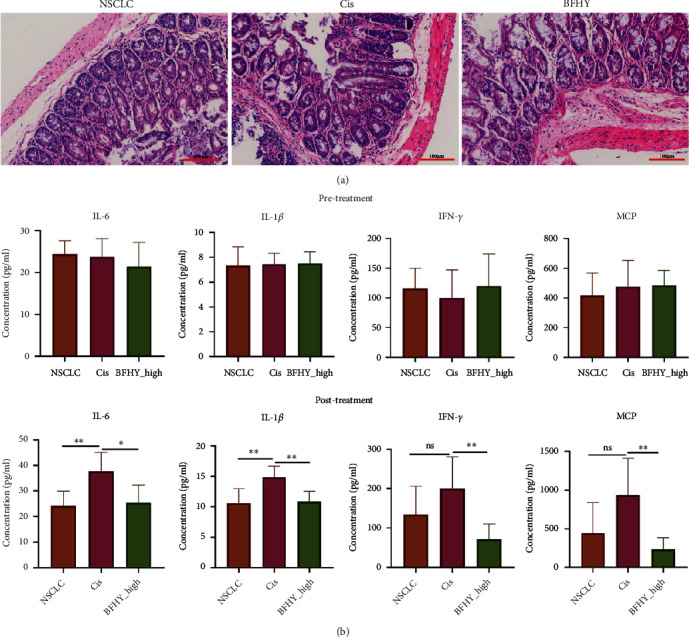
BFHY could against the intestine toxicity induced by cisplatin. (a) H&E staining of cecum sample at 200×. (b) ELISA analysis of IL-6, IL-1*β*, MCP, and IFN-*γ* in pre-treatment serum and post-treatment cecum contents. The number of cecum tissue is there and cecum contents are six. Statistical significance was determined by multiple *t* test, ∗*p* < 0.05, ∗∗*p* < 0.01.

Published studies had implicated that chemotherapy caused intestinal toxicity, and TCMs were proven to be against toxicity. To observe it, we analyzed the cecum tissue. H&E analysis revealed that mucosal epithelia were intact in NSCLC group; on the contrary, the mucosa was seriously damaged, and a larger number of inflammatory cells were infiltrated in Cis group. When combined with BFHY, the histological damage of cecum was relieved (Figure 2(a)). Pre-treatment serum expression of inflammatory cytokines showed consistent baseline levels in all groups. Post-treatment expression of inflammatory cytokines in cecum contents showed that cisplatin treatment-induced IL-6, IL-1*β*, MCP, and IFN-*γ* significantly increased compared with NSCLC group, and the situation was reversed by BFHY (Figure 2(b)). These histological and ELISA results indicated that BFHY could relieve cecum damage and inflammation caused by chemotherapy in NSCLC.

### 3.3. Cisplatin and BFHY Treatment Alters Gut Microbial in Mice Models of Lung Cancer

**Figure 3:**
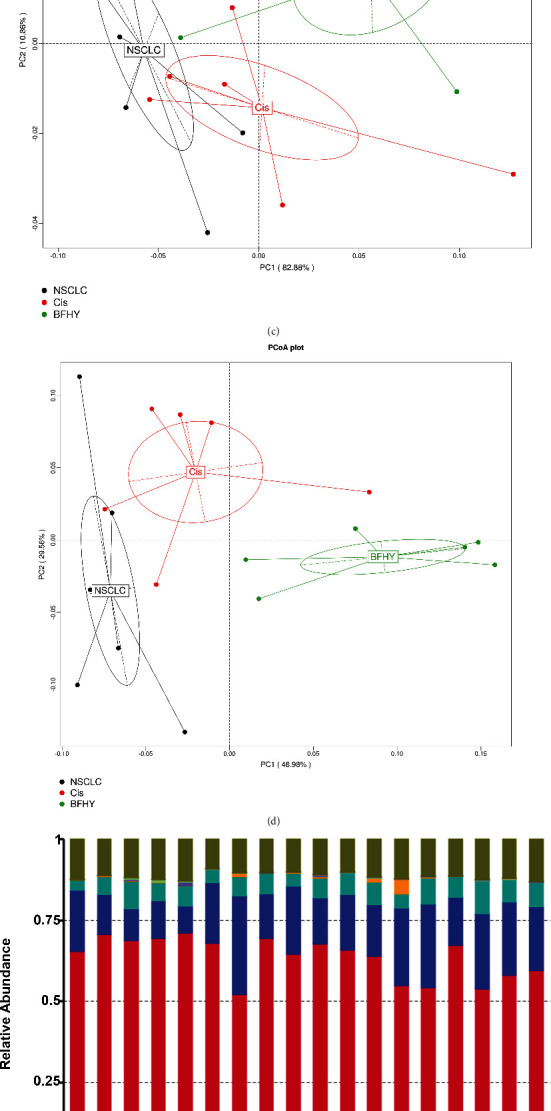
The gut microbial profile after cisplatin and BFHY treatment. (a) The numbers of sequenced genes in three groups by box chart. (b) Gene numbers between three groups by Venn diagram. (c and d) Principal coordinates analysis (PCoA) revealed that the beta diversity for all groups was exhibited with Bray–Curtis distance in phyla and genus. Each point represents a sample and the samples of the same group are represented by the same color. (e and f) Percent abundance of microbial in phyla and genus. NSCLC: non-small cell lung cancer; Cis: cisplatin; BFHY: Bu Fei Hua Yu. *n* = 6/group.

Recent investigations indicated that chemotherapy significantly alters gut microbiota; further, TCMs can be digested by gut microbiota, which may further affect microbiota composition. We performed metagenomic sequencing on fecal samples at the end point of experiment. Total gene numbers were calculated in three groups; there were no significance between the groups; however, we found that the average number of genes in Cis group was lower than NSCLC and BFHY groups; and Venn diagram showed that the common genes between the groups account for the majority (Figures 3(a) and 3(b)). Principal coordinates analysis (PCoA) in phylum and genus level indicated that there was a certain degree of distinction between different groups, among which, the differences between individuals in the Cis group were relatively large (Figures 3(c) and 3(d)). In phylum level, we found that distributions of top 10 in different samples were *Bacteroides*, *Firmicutes*, and *Proteobacteria*, which were similar to the generally reported mice gut microbiota composition (Figure 3(e)). In genus level, the top 10 distributions of different samples were *Bacteroides*, *Blautella*, *Parabacteroides*, *Prevotella*, etc. (Figure 3(f)).

### 3.4. Differences in Bacterial Communities between Different Efficacy Groups

**Figure 4:**
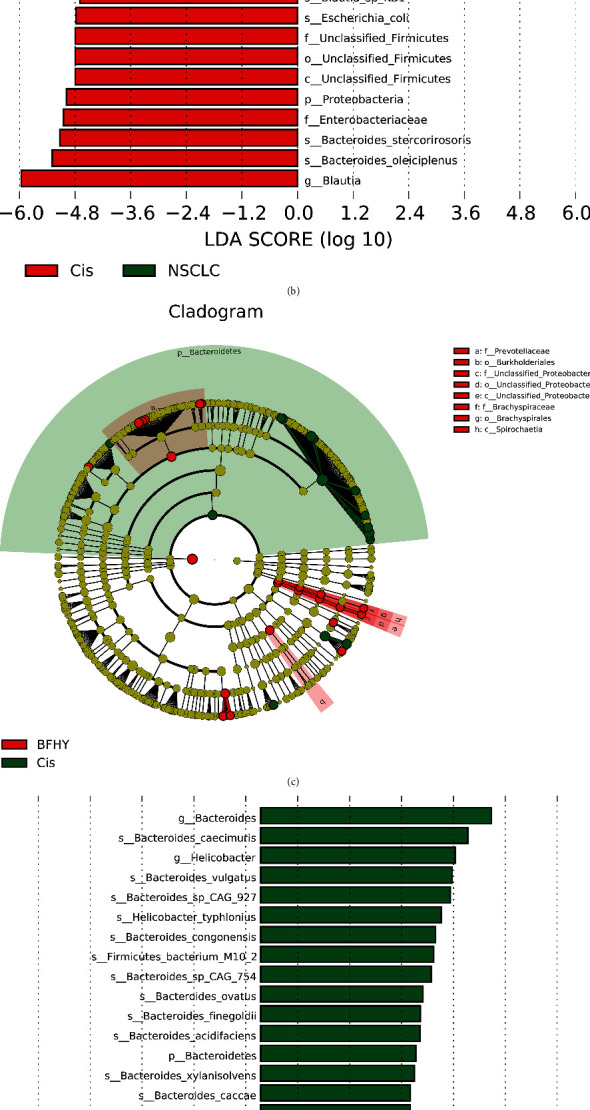
Differences in the microbiomes of mice with cisplatin and combination with BFHY. LEfSe analysis was used to distinguish the differential microbiome between two groups. (a and c) Phylogenic relationship of tax, which is significantly different between NSCLC and Cis groups (a) and between Cis and BFHY (c). The different-colored nodes represent microbial populations that were significantly enriched in the corresponding groups and that showed significant differences between the groups. The yellow nodes indicate microbial groups that showed no significant differences between two groups. The circles going from the inside to the outside represent the phylum, class, order, family, and genus. (b and d) LDA was performed, and only the microbiota with LDA scores of >4 is shown. (e) Differential genus between NSCLC, Cis, and BFHY combination by heatmap.

To identify the most differentially abundant bacteria in cisplatin and BFHY-treated mice, we performed LEfSe analysis and selected biomarkers with LDA >4. The results showed that the microbiomes relative abundance were changed significantly between the three groups (Cis-vs-NSCLC and BFHY-vs-Cis) from phylum to species, and we focus on genus level in further study. *f_Enterobacteriaceae*, *c_Erysipelotrichia,* and *o_Erysipelotrichales* were upregulated after cisplatin treatment (Figures 4(a) and 4(b)). After combination with BFHY, *g_Bacteroides* and *g_Helicobacter* were decreased. *g_Klebsiella*, *g_Unclssified_Proteobacteria,* and *g_Unclssified_Clostridiates* were increased (Figures 4(c) and 4(d)).  Figure 4(e) showed the above difference in genus in the three groups by heatmap, and we found that at the genus level, *Hungatella*, *Klebsiella*, *Blautia*, and *Escherichia* abundance were increased after cisplatin and BFHY treatment, in which *Blautia* and *Escherichia* abundance increased significantly in Cis group compared with NSCLC group. *Parabacteroides* abundance was decreased significantly in Cis group compared with NSCLC group. Moreover, *Bacteroides* abundance was increased significantly after cisplatin treatment, and BFHY combination treatment reversed the state.

### 3.5. Differences in Bacterial Community Function between Different Efficacy Groups

**Figure 5:**
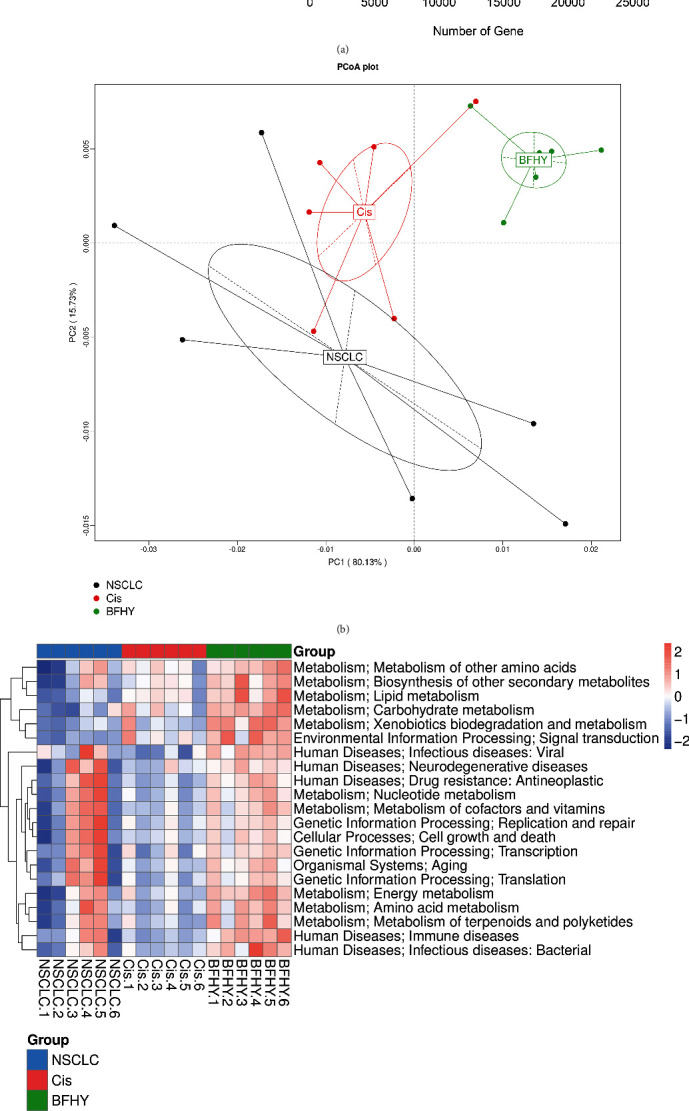
Functional analysis of the gut microbiota in three groups. The Unigenes from metagenomic sequencing were blasted to KEGG database and filtered for further analysis. (a) The bar plot of the number of Unigenes annotated in KEGG database, the number on the bar graph represents the number of Unigenes on the note. (b) The PCA analysis of KEGG function abundance of all samples in three groups. Each point represents a sample, and the samples of the same group are represented by the same color. (c) The cluster analysis of KEGG function relative abundance of all samples in three groups. (d) The box plot of functional difference between group. The Metastats method was used to obtain the *p* value. ∗*p* < 0.05, ∗∗*p* < 0.01.

To predict functional composition profiles in all samples of three groups, we compared Unigenes with KEGG functional database by using the DIAMOND software.  Figure 5(a) showed the annotated gene numbers, and we found that the most mounts of genes were involved in “Metabolism” and mainly included “nucleotide metabolism”, “carbohydrate metabolism”, “amino acid metabolism”, “energy metabolism”, and “metabolism of cofactors and vitamins”. Moreover, “signal transduction” and “translation” were also enriched. PCA analysis showed that there was a certain degree of distinction between three groups of the function, among which, the sample heterogeneity was the largest in NSCLC group (Figure 5(b)). To study the differential function between groups, we analyzed the KEGG level 2 categories by Metastats method, and found several functions were decreased after cisplatin treatment and recovered by adding BFHY, such as “nucleotide metabolism”, “immune diseases”, “energy metabolism”, and “aging” (Figure 5(c)).  Figure 5(d) showed top 6 differential functions, and we found all of them were slightly decreased in Cis group compared with NSCLC group and increased significantly in BFHY combination groups, which included “cell growth and death”, “replication and repair”, “translation”, “amino acid metabolism”, “metabolism of cofactors and vitamins”, and “nucleotide metabolism”.

## 4. Discussion

BFHY is a clinical experience formula for treating lung cancer; our previous clinical study showed that combination of BFHY with chemotherapy can significantly improve the clinical efficacy and quality of life by reducing intestinal toxicity [7, 8]. Reported studies showed that chemotherapy can lead to intestinal inflammation and decrease in the gut microbiota diversity [22]; however, whether gut microbiota involved in BFHY's efficacy is not clear. In our study, the gut microbiota of the mice model of lung cancer, which received combination of BFHY with cisplatin treatment, was profiled. We found that BFHY enhances the ability of cisplatin to kill tumor and relieved intestinal toxicity by decreasing the inflammatory factors. Metagenomic sequencing results showed that the compositions of gut microbiota changed significantly after combination with BFHY, in which, *g_Bacteroides* and *g_Helicobacter* were decreased and *g_Klebsiella*, *g_Unclssified_Proteobacteria*, and *g_Unclssified_Clostridiates* were increased. Bacterial communities function analysis showed that “cell growth and death”, “replication and repair”, “translation”, “amino acid metabolism”, “metabolism of cofactors and vitamins”, and “nucleotide metabolism” were significantly increased.

Consistent with our previous clinical research results [7, 8], when combined with BFHY, the tumor growth and intestinal toxicity both were restricted. In fact, TCMs, which are applied as an adjuvant therapy to treat cancer, is not a fresh view and has approved to improve efficacy. Taohong Siwu Decoction combined with neoadjuvant chemotherapy reduces tumor lymphatic vessels and tumor angiogenesis in blood stasis breast cancer [23]. Adjuvant chemotherapy combined with TCM, including benefiting qi recipe, benefiting yin recipe, benefiting qi and yin recipe, and detoxication and resolving masses recipe, is better than adjuvant chemotherapy alone for postoperative NSCLC patients [24]. Yang et al. showed that the PFS (progression free survival) was better in NSCLC patients of whole population treated with gefitinib plus TCM than with gefitinib only [25]. In addition, Tang et al. found that *Jian Pi Li Gan Decoction* could improve long-term survival of hepatocellular carcinoma patients through increasing the success of radiofrequency ablation treatment [26]. Moreover, TCM alleviates the symptoms and adverse reactions caused by chemotherapy, radiotherapy, or targeted therapy [27]. All those studies focused on TCM application in antitumor mechanisms; however, the mechanisms of intestinal damage relive are not clear. There is evidence implicated the gut microbiota in influencing response, intestinal toxicity and administration of chemotherapeutics can damage the diversity and health of gut microbiota [13, 28, 29]. Acupuncture is an important complementary therapy used in cancer treatment, Xu et al. found that acupuncture treatment can inhibit the development of osteosarcoma by regulating intestinal microbiome [30]. What's more, in a randomized, placebo-controlled, blind trial, Wang et al. observed that Shenhuang plaster application could relieve chemotherapy-induced gastrointestinal toxicity in breast cancer patients [31]. Additionally, gut microbiota promotes the maintaining of health and acts as adjuvants in the treatment of cancer [32]. In our own study, we also observed a significant difference in composition of gut microbiota in response to cisplatin in the mice model of lung cancer. The above results revealed that cisplatin treatment could induce intestinal damage and gut microbiota dysbiosis.

We explored whether BFHY's protective role against cisplatin treatment-induced injury is related to gut microbiota. Heatmaps revealed that *Bacteroides* abundance was increased significantly after cisplatin treatment, and BFHY combination treatment reverses the state. Jang et al. found that *Bacteroides* was more abundant in non-complete response than complete response patients after concurrent chemoradiation and indicates a strong factor with poor response [33]. Okubo et al. also found that the high abundance of *Bacteroides* genus was directly related to fear of cancer recurrence in breast cancer survivors [34]. We, therefore, hypothesized that BFHY's protective role maybe associated with decreasing of *Bacteroides* in mice model of lung cancer.

Our function analysis revealed that “cell growth and death”, “replication and repair”, “translation”, “amino acid metabolism”, “metabolism of cofactors and vitamins”, and “nucleotide metabolism” were increased significantly after combination with BFHY. “Amino acid metabolism” plays a vital role in the occurrence of chemoresistance. Obrist et al. found that glutamine was majorly utilized for nucleotide biosynthesis in cisplatin-resistant NSCLC cells [35]. In addition, the cisplatin resistance in bladder cancer was reported related to argininosuccinate synthase 1 and spermidine/spermine N1-acetyltransferase-mediated amino acid metabolism [36]. Suppression of thymidine phosphorylase could enhance the drug responsiveness of gastric cancer cell to 5-fluorouracil [37], and the results indicated that “nucleotide metabolism” was also closely associated with chemotherapy. Other metabolic pathway is also reported to have an association with chemoresistance. BFHY's ability to enhance tumor killing may be achieved through metabolic interference, which has great potential in tumor adjuvant therapy.

There are limitations in our current study. First, we just analyzed that the gut microbiota profile at the end of the experiment in different treatments in one cell line A549, more cell lines, and microbiota profiles in different times (such as before the start of the treatments and before the injection of the tumor cells) are needed in the further study. Second, our results may not accurately identify the relationship between intestinal toxicity and the decreased abundance of *Bacteroides*; in addition, the functions of *Bacteroides* are also not further studied. Thirdly, the role of BFHY combination chemotherapy in reducing *Bacteroides* abundance remains to be elucidated.

## 5. Conclusion

In this study, we revealed differential microbial communities and functions in terms of therapeutic response after combination of BFHY with mice of lung cancer model. Gut microbiomes, such as *Bacteroides*, were decreased, and its relation to intestinal toxicity remains further research. Our study provides new strategies to prevent chemotherapy-induced intestinal toxicity in microbiota.

## Data Availability

The data that support the findings of this study are available from the corresponding author upon reasonable request.

## Abbreviations

BFHY: Bu Fei Hua Yu
NSCLC: Non-small cell lung cancer
TCMs: Traditional Chinese medicines
TSA: Trichostatin A
LEfSe: Linear discriminant analysis effect size
LDA: Linear discriminant analysis.
